# Decomposition analysis of anxiety symptom differences between urban and rural Chinese university students: cross-sectional study

**DOI:** 10.3389/fpsyt.2025.1639959

**Published:** 2025-09-18

**Authors:** Yusupujiang Tuersun, Yuying Xie, Qingping Zhou, Yao Yu, Wenyu Wang, Chenxi Wang, Siyuan Liu, Yuyao Song, Zhenning Liang, Yi Qian

**Affiliations:** ^1^ School of Health Management, Southern Medical University, Guangzhou, China; ^2^ Grade III, Class A Hospital Accreditation Office, Shenzhen Longhua Maternity and Child Healthcare Hospital, Shenzhen, China; ^3^ Nanfang Hospital, Southern Medical University, Guangzhou, China; ^4^ Institute of Health Management, Southern Medical University, Guangzhou, China; ^5^ School of Public Health, Southern Medical University, Guangzhou, China; ^6^ The Seventh Affiliated Hospital, Sun Yat-sen University, Shenzhen, China

**Keywords:** anxiety symptoms, Fairlie decomposition model, college students, China, rural and urban

## Abstract

**Background:**

Anxiety is a prevalent psychological disorder characterized by excessive worry and fear, which significantly impacts the mental health of university students. Anxiety symptoms are often misunderstood, leading to delays in seeking help. This issue is particularly notable among college students, who may experience anxiety due to academic pressures, societal expectations, and family issues. This study explores anxiety symptoms among urban and rural Chinese university students, aiming to analyze the differences and contributing factors. The primary goal of this study is to analyze the differences in anxiety symptoms between urban and rural Chinese university students and to decompose these differences into the contributions of various individual and socio-economic factors using the Fairlie decomposition model.

**Methods:**

A cross-sectional survey was conducted between January and February 2023, involving 7,230 valid questionnaires from undergraduate students across ten provinces in China. The Generalized Anxiety Disorder 7 (GAD-7) scale was used to assess anxiety symptoms. Demographic characteristics, academic performance, lifestyle factors, depressive symptoms (Patient Health Questionnaire-9; PHQ-9), and eHealth (SeHealth Literacy Scale) literacy were controlled for as covariates. The Fairlie decomposition model was applied to examine the contributing factors to the observed differences in anxiety symptoms between urban and rural students.

**Results:**

The study found that 38.91% of students reported experiencing anxiety symptoms. The prevalence was higher among rural students (40.2%) compared to urban students (36.8%). Significant factors contributing to this difference included depressive symptoms (51.07% contribution), exercise habits (7.07% contribution), and family income levels. Rural students were found to be at higher risk for anxiety symptoms, with those from lower-income families and those lacking exercise habits exhibiting greater anxiety.

**Conclusion:**

The results demonstrate a significant difference in anxiety symptoms between urban and rural college students in China. The disparity is mainly influenced by depressive symptoms, exercise habits, and family income. The findings suggest the need for tailored mental health interventions for rural students, with a focus on improving access to resources, promoting physical activity, and addressing socio-economic factors. The study underscores the importance of considering urban-rural differences in the development of effective mental health strategies for university students.

## Background

Anxiety is a psychological disorder characterized by excessive worry and fear, often accompanied by significant physical and behavioral responses (1). Anxiety is characterized by persistent nervousness, hypervigilance to potential threats, and impaired functioning of daily living due to avoidance or somatic symptoms (2, 3). Studies have shown that many people with anxiety disorders do not seek professional intervention in time due to misunderstanding of symptoms or stigma (4), and this phenomenon is particularly prominent among college students, which significantly affects their physical and mental health and development potential (5, 6).

The college years are a critical period for the development of mental health. Physical and mental health at this special stage is not only about academic achievement and interpersonal development, but also about career trajectory and quality of life for decades to come. The Chinese education system, which is rooted in the Confucian cultural tradition, places the pressure on the expectations of parents and the future career development of society, including academic requirements, parental expectations, and peer pressure (7). All these factors contribute to increasing the psychological pressure of college students and lead to various mental health problems. According to the research, the physical fitness of college students is showing a continuous downward trend, the incidence of sub-health is relatively high, and the overall mental health level is not optimistic (8, 9).

College student anxiety often presents with uncontrollable tension, catastrophic expectations regarding future events, irritability, or emotional out-of-control, and in some patients a strong fear of a specific scenario (e.g., socializing, exams) (10). Anxiety-led attention distraction and procrastination can easily lead to a decline in college students’ academic performance, failure in exams and even suspension of school (11–13). Social anxiety encourages students to avoid group activities, hinders the establishment of interpersonal support networks, exacerbates loneliness and self-denial, and may induce comorbid depression (14, 15). Long-term stress weakens immunity, increases the risk of physical conditions such as hives and irritable bowel syndrome, and may trigger eating disorders such as binge eating or anorexia. College students’ anxiety not only exacerbates family financial pressure (such as repeated medical treatment and school suspension costs), but also consumes campus psychological counseling resources and reduces the overall educational effectiveness (16). Anxiety-related absenteeism, health expenditures, and loss of productivity further pose challenges to psychosocial health services (17, 18).

The influencing factors of anxiety among college students focus on personal factors such as gender, major, academic performance, place of birth (urban or rural), family factors such as income and number of siblings, and their own health (19–21).With the promotion of plans and policies such as the “Healthy China Strategy”, the mental health problems of college students have received more and more attention, and there is an urgent need to carry out intervention research on college students’ anxiety (22, 23). However, the existing research on the determinants of college students’ anxiety still needs to be further deepened, and there is a lack of detailed data analysis and stratified comparison of students with different characteristics, as well as a systematic comparison of the effects of differences. This prevents the provision of a concrete and reliable empirical basis for intervention research and subsequent policy development. In this study, we used the Fairlie decomposition model to understand the factors influencing differences in anxiety symptoms among college students in urban and rural areas. Participants were grouped according to their urban or rural situation; Demographic, social, personal lifestyle, and depressive symptoms were used as covariates. This study aims to analyze the differences in anxiety symptoms among urban and rural college students, and decompose these differences into the contributions of different individual characteristic factors. The purpose is to investigate the root causes of this inequality and provide theoretical evidence for promoting health equity in both urban and rural areas of China.

## Methods

### Data source

This cross-sectional research study utilized an online survey platform (https://www.wjx.cn/) to administer an anonymous digital questionnaire through convenience sampling approach between January and February 2023. Participants were recruited from undergraduate students across ten Chinese provinces and municipalities, including Guangdong, Shanghai, and Jiangsu, representing diverse academic disciplines including economics, medicine, management, and literature. The study was approved by the Biomedical Ethics Committee of Southern Medical University (Southern Medical University Ethics Review Board (2023) No. 46). Written informed consent was secured from all subjects prior to survey initiation. The web-based Questionnaire Star system facilitated questionnaire distribution, collection, and encrypted data storage. Implemented restricted access protocols and tamper-evident safeguards ensured data security. Compliance with national regulations was maintained throughout the study. Participant anonymity will be preserved in all public disclosures. Final datasets were converted into EpiData format for statistical processing.

**Figure 1:**
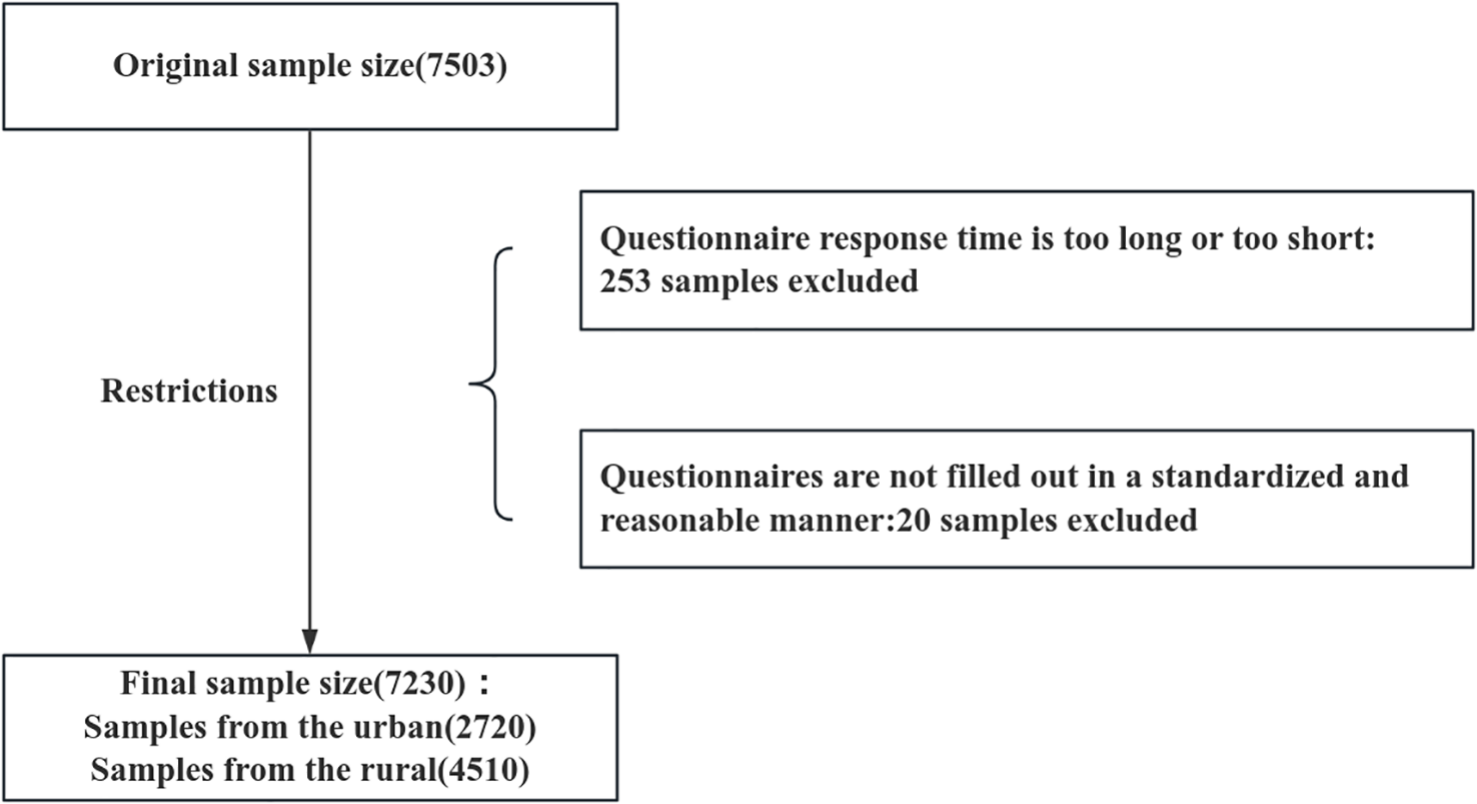
Participant screening flowchart.

To ensure the accuracy and reliability of the data, the questionnaire included two verification questions, a repetitive question and a general knowledge question (where is the capital of China). If a respondent answered incorrectly to either question, the questionnaire was considered invalid and excluded from further analysis. A total of 7,503 questionnaires were collected. Following screening, 253 questionnaires with anomalous response times and 20 questionnaires with irregular completion were excluded, leaving 7,230 valid questionnaires, representing an effective recovery rate of 96.3%. The subjects included in this study were required to meet the following criteria: Undergraduate students; Have normal cognitive function and be able to complete the questionnaire independently. The following exclusion criteria were applied: Incorrect responses to the verification questions. The exclusion process is illustrated in **Figure 1**.

### Anxiety symptoms

The GAD-7 scale was utilized to evaluate anxiety symptom prevalence and severity in the research cohort. This instrument consists of seven questions, each scored on a 4-point Likert scale ranging between 0 and 3. The scoring criteria are defined as: 0 = “never,” 1 = “occasionally,” 2 = “frequently,” and 3 = “daily.” Scores below 5 suggest no significant anxiety symptoms, while scores ≥5 indicate clinically relevant anxiety (24). During this investigation, the GAD-7 demonstrated exceptional internal consistency with a Cronbach’s alpha value of 0.944, with 0.942 for the rural university student group and 0.947 for the urban university student group.

### Grouping variables

Respondents were categorized into rural or urban groups depending on the type of household they reported during the survey.

### Covariates

In order to obtain more reliable results, a range of potential confounding variables were controlled for. These included demographic characteristics, sociological characteristics, personal lifestyles, depressive symptom status and eHealth literacy levels that have been previously utilized in other studies of anxiety symptom status (25, 26).

### Demographic characteristics

The participants were classified according to two variables: gender (male or female) and personal religious belief (yes or no).

### Sociological characteristics

The respondents were classified into four groups based on their academic performance, determined by their class ranking: below 25%, 25%-50%, 50%-75%, and above 75%. Furthermore, monthly per capita household income was divided into four categories: less than 2,500 yuan, 2,500-5,000 yuan, 5,000-10,000 yuan, and above 10,000 yuan.

### Personal lifestyle

The variables of exercise, smoking, and alcohol use were dichotomized into a binary format, with responses of “yes” or “no” assigned to each item. The term “exercise” was defined as engaging in physical activity on three or more occasions over the past month, with each session lasting at least 30 minutes. Moreover, the use of tobacco products and alcoholic beverages (including distilled spirits, beer, wine, and rice wine) in the past month was recorded as a dichotomous variable (yes or no).

### Depressive symptom

The PHQ-9 was employed to assess depressive symptoms among the participants. The PHQ-9 is comprised of nine items and employs the same scoring method as the GAD-7. A score below 5 indicates the absence of depressive symptoms, whereas a score of 5 or higher suggests the presence of depressive symptoms (27). In this study, the PHQ-9 exhibited a Cronbach’s alpha coefficient of 0.931. In this study, depressive symptoms were included as one of the five explanatory domains used to understand group-level differences in anxiety symptoms. We emphasize that the PHQ-9 variable was not used to imply a causal pathway from depression to anxiety, but to estimate its statistical contribution to the observed disparity in anxiety symptoms between urban and rural university students.

### eHealth literacy

The research employed the Chinese adaptation of the eHealth Literacy Scale (eHEALS) to measure university students’ eHealth literacy capabilities. Originally developed by Norman, Skianer, and colleagues in 2016, this validated instrument has been extensively used in digital health competency evaluations (26). Multiple studies across varied demographic groups have reported consistently elevated Cronbach’s alpha values for the eHEALS instrument, generally falling between 0.84 and 0.94. These findings confirm the measurement tool’s strong internal consistency and reliability across different population samples. The scale is composed of eight items, which are classified into three categories of competencies related to the use of online health information and services: application competencies (entries 1-5), judgmental competencies (entries 6-7), and decision-making competencies (entry 8). The scale employs a five-point Likert scale, with scores ranging from one to five. The total score for the eight items ranges from 8 to 40 points. A higher total score is indicative of a higher level of eHealth literacy. In this study, the widely recognized cut-off score of 32 was used to categorize the eHealth literacy of urban and rural university students into two categories: “qualified” (≥32 points) and “unqualified” (<32 points) (28, 29). The overall Cronbach’s alpha coefficient for this study was 0.974.

### Statistical analysis

The fundamental demographic characteristics and lifestyles of the study population were analyzed in a descriptive manner. Continuous variables were described by the mean ± standard deviation, while categorical variables were described by the percentage. A chi-square test was employed to analyze the distribution of anxiety symptom among urban and rural university students. Subsequently, a binary logistic regression model was constructed in this study to identify the main determinants for determining the anxiety symptom among urban and rural university students. The outcome variable was defined as a binary categorization of anxiety symptoms (yes/no), while the independent variables were selected from five domains: demographic characteristics, sociological characteristics, personal lifestyle, depressive symptom and eHealth literacy levels. Initially, these variables were screened by univariate analysis (retention criteria: *P*<.05), and subsequently, they were included in the multivariate model. Through this process, statistically significant predictors (*P*<.05) were retained in the final model. The aforementioned statistical analyses were conducted using IBM SPSS Statistics 23 software. Ultimately, the Fairlie model was employed to examine the factors contributing to the discrepancies in anxiety symptoms between urban and rural university students. The analysis was conducted using Stata MP 18.0 software. The level of statistical significance was set at 0.05.

### Fairlie decomposition model

In this study, we applied a multivariate Fairlie decomposition analysis (FDA) based on a binary regression model. FDA is one of the decomposition techniques used in multivariate models to quantify the contribution of predicted differences between two groups to outcome variables. The method is an extension of the Blinder-Oaxaca decomposition analysis, which has been widely criticized for its inefficiency in handling the logit and probit model. The FDA was developed specifically for nonlinear regression models, including logit and probit models (30). The FDA identifies the contribution of independent variables to explaining differences between groups by calculating the change in the mean predicted probability resulting from substituting one independent variable at a time in one group (e.g., Group A - rural university students) while holding other variables constant in the other group (e.g., Group B - urban university students). The Fairlie decomposition technique works by limiting the predicted probability to between 0 and 1.Studies have shown that the FDA can better quantify the contribution and significance levels of different variables for nonlinear regression models (31, 32). Given that the dependent variable was a dichotomous variable, we employed Fairlie’s nonlinear decomposition to decompose the discrepancies in university students’ eHealth literacy into the contributions of various factors. In this context, the Fairlie decomposition method was chosen over the traditional Oaxaca-Blinder approach for several reasons. First, our outcome variable—presence or absence of anxiety symptoms—is binary, which renders linear decomposition methods such as Oaxaca-Blinder less suitable. The Fairlie method was specifically developed to handle non-linear models like logistic regression, making it a more appropriate choice for our analysis. Second, we acknowledge that the Fairlie decomposition is sensitive to the ordering of covariates, which may introduce variability into the estimated contributions. To mitigate this limitation, we followed Fairlie’s recommendation and conducted 100 randomized replications of the decomposition process using Stata MP 18.0, averaging the results to reduce the impact of variable ordering. This approach has been demonstrated in previous studies to enhance the stability and reliability of decomposition results. Third, the Fairlie method has been widely adopted in public health and economics research to examine disparities in binary outcomes, including studies of racial disparities in healthcare access, health insurance coverage, and mental health conditions. Its strong theoretical foundation and demonstrated applicability to health inequality studies support its use in the present research (33). As outlined by Fairlie (34), the decomposition of the nonlinear equation can be expressed as follows:


(1) $${\bar{Y}}_{e}-{\bar{Y}}_{w}=[\sum_{i=1}^{N^{e}}\frac{F(X_{i}^{e}\beta^{e})}{N^{e}}-\sum_{i=1}^{N^{w}}\frac{F(X_{i}^{w}\beta^{e})}{N^{w}}]+[\sum_{i=1}^{N^{w}}\frac{F(X_{i}^{w}\beta^{e})}{N^{e}}-\sum_{i=1}^{N^{w}}\frac{F(X_{i}^{w}\beta^{w})}{N^{w}}]$$

The symbols ${\bar{Y}}_{e}$ and ${\bar{Y}}_{w}$ represent the mean probabilities of the two binary outcomes. The F is employed to indicate the cumulative distribution function of the logistic distribution. Ye-Yw represents the total difference in the differences between the two groups. The N^e^ and N^w^ represent the sample sizes of the two samples. The first term in parentheses in equation (1) represents the portion of the gap attributable to differences in observed characteristics, while the second term denotes the portion of the gap due to differences in estimated coefficients.

## Results

### Basic characteristics of the study subjects

The study included a total of 7,230 samples. The average GAD-7 score of college students was 3.72±4.001 (**Table 1**). A score of 5 or less indicates no anxiety symptoms, and a score of 5 or more indicates the presence of anxiety symptoms. The results of **Table 2** showed that 4417 college students (61.09%) had no anxiety symptoms, and 2813 college students (38.91%) had anxiety symptoms. The results of the chi-square test in **Table 2** show that there are significant differences in the distribution of the eight covariates between urban and rural college students. These covariates included gender, religion, class ranking, monthly per capita household income, exercise, drinking, PHQ-9 score, and eHealth literacy score. However, no significant differences were found in the distribution of smoking factors.

**Table 1:** GAD-7 score for university students.

Item	Overall(x ± s)	Rural(x ± s)	Urban(x ± s)
Q1:Feeling nervous, anxious, or on edge	0.70 ± 0.681	0.71 ± 0.663	0.68 ± 0.710
Q2:Not being able to stop or control worrying	0.56 ± 0.675	0.57 ± 0.662	0.55 ± 0.695
Q3:Worrying too much about different things	0.57 ± 0.683	0.58 ± 0.670	0.55 ± 0.703
Q4:Trouble relaxing	0.52 ± 0.653	0.53 ± 0.638	0.52 ± 0.677
Q5:Being so restless that it’s hard to sit still	0.43 ± 0.631	0.44 ± 0.622	0.42 ± 0.646
Q6:Becoming easily annoyed or irritable	0.52 ± 0.661	0.53 ± 0.643	0.51 ± 0.690
Q7:Feeling afraid as if something awful might happen	0.41 ± 0.636	0.42 ± 0.619	0.41 ± 0.663
Total score	3.72 ± 4.001	3.77 ± 3.893	3.63 ± 4.172

**Table 2:** Distribution of the variables in rural and urban respondents.

Variable	Urban [n(%)]	Rural [n(%)]	*x*2	*P*
GAD-7			8.135	0.002
≥5	1001 (36.8)	1812 (40.2)		
<5	1719 (63.2)	2698 (59.8)		
Gender			58.356	<.001
Male	1055 (38.8)	1355 (30.0)		
Female	1665 (61.2)	3155 (70.0)		
Religious belief			18.783	<.001
Yes	110 (4.0)	291 (6.4)		
No	2610 (96.0)	4219 (93.6)		
Class ranking			9.832	0.02
<25.0%	781 (28.7)	1217 (27.0)		
25.0%~<50.0%	1026 (37.7)	1623 (36.0)		
50.0%~<75.0%	658 (24.2)	1233 (27.3)		
≥75.0%	255 (9.4)	437 (9.7)		
Monthly per capita household income			10002.18	<.001
<2 500RMB	219 (8.1)	1158 (25.7)		
2 500~<5 000RMB	852 (31.3)	2163 (48.0)		
5 000~<10 000RMB	1030 (37.9)	952 (21.1)		
≥10 000RMB	619 (22.7)	237 (5.2)		
Exercise			28.08	<.001
Yes	2016 (74.1)	3078 (68.3)		
No	704 (25.9)	1432 (31.7)		
Smoking			0.008	0.93
Yes	84 (3.1)	141 (3.1)		
No	2636 (96.9)	4369 (96.9)		
Drinking			49.534	<.001
Yes	549 (20.2)	626 (13.9)		
No	2171 (79.8)	3884 (86.1)		
PHQ-9			4.42	0.04
≥5	1222 (44.9)	2141 (47.5)		
<5	1498 (55.1)	2369 (52.5)		
eHealth literacy			159.781	<.001
≥32	1422 (52.3)	1673 (37.1)		
<32	1298 (47.7)	2837 (62.9)		

Class ranking :academic achievement, with students categorized based on their relative ranking within their class.

eHEAL, SeHealth Literacy Scale; GAD-7, 7-item Generalized Anxiety Disorder; PHQ-9, 9-item Patient Health Questionnaire.

### Comparison of variables’ distribution

**Table 3** shows the relationship between the distribution of covariates among urban and rural college students and the presence of anxiety symptoms. The data indicates that certain covariates exhibit similar characteristics among students assessed for the presence or absence of anxiety symptoms. The following factors were identified as being associated with anxiety symptoms in college students: gender, religious belief, monthly per capita household income, drinking, and eHealth literacy level. Although results for both the absence and presence groups are presented in **Table 3** to provide a more comprehensive picture of the population, our interpretation in the Results and Discussion sections primarily focuses on the presence of anxiety symptoms, as this represents the main objective of the study.

**Table 3:** Distribution of variables among survey respondents the presence or absence of anxiety symptoms.

Variable	The absence of anxiety symptoms				The presence of anxiety symptoms			
	Urban	Rural	*x*2	*P*	Urban	Rural	x2	P
	[n(%)]	[n(%)]			[n(%)]	[n(%)]		
Gender			41.635	<.001			14.225	<.001
Male	726 (42.2)	881 (32.7)			329 (32.9)	474 (26.2)		
Female	993 (57.8)	1817 (67.3)			672 (67.1)	1338 (73.8)		
Religious belief			14.253	<.001			4.772	0.029
Yes	64 (3.7)	171 (6.3)			46 (4.6)	120 (6.6)		
No	1655 (96.3)	2527 (93.7)			955 (95.4)	1692 (95.4)		
Class ranking			12.278	0.006			0.554	0.907
<25.0%	486 (28.3)	702 (26.0)			295 (29.5)	515 (28.4)		
25.0%~<50.0%	669 (38.9)	981 (36.4)			357 (35.7)	642 (35.4)		
50.0%~<75.0%	411 (23.9)	770 (28.5)			247 (24.7)	463 (25.6)		
≥75.0%	153 (8.9)	245 (9.1)			102 (10.2)	192 (10.6)		
Monthly per capita household income			631.085	<.001			369.034	<.001
<2 500RMB	115 (6.7)	622 (23.1)			104 (10.4)	536 (29.6)		
2500~5000RMB	515 (30.0)	1326 (49.1)			337 (33.7)	837 (46.2)		
5000~10000RMB	678 (39.4)	584 (21.6)			352 (35.2)	368 (20.3)		
≥10 000RMB	411 (23.9)	166 (6.2)			208 (20.8)	71 (3.9)		
Exercise			24.497	<.001			3.742	0.053
Yes	1354 (78.8)	1946 (72.1)			662 (66.1)	1132 (62.5)		
No	365 (21.2)	752 (27.9)			339 (33.9)	680 (37.5)		
Smoking			0.345	0.557			0.379	0.538
Yes	47 (2.7)	82 (3.0)			37 (3.7)	59 (3.3)		
No	1672 (97.3)	2616 (97.0)			964 (96.3)	1753 (96.7)		
Drinking			24.814	<.001			27.591	<.001
Yes	311 (18.1)	341 (12.6)			238 (23.8)	285 (15.7)		
No	1408 (81.9)	2357 (87.4)			763 (76.2)	1527 (84.3)		
PHQ-9			0.056	0.813			0.050	0.824
≥5	308 (17.9)	491 (18.2)			914 (91.3)	1650 (91.1)		
<5	1411 (82.1)	2207 (81.8)			87 (8.7)	162 (8.9)		
eHealth literacy			97.546	<.001			55.287	<.001
≥32	992 (57.7)	1146 (42.5)			430 (43.0)	527 (29.1)		
<32	727 (42.3)	1152 (57.5)			571 (57.0)	1285 (70.9)		

eHEAL, SeHealth Literacy Scale; GAD-7, 7-item Generalized Anxiety Disorder; PHQ-9, 9-item Patient Health Questionnaire.

### Logistic model results

**Table 4** shows the logical modeling results of having any anxiety symptoms among urban and rural college students. Among urban college students, those from households with a monthly per capita income of 5,000–10,000 RMB were significantly less likely to report anxiety symptoms compared with those from the lowest income group (<2,500 RMB) (OR=0.585, 95% CI=0.380–0.901, p<0.05). Conversely, exercise (NO, OR = 1.342) was identified as a protective factor. Among rural college students, smoking (NO, OR = 0.572) and PHQ-9 scores (5, OR = 0.022) were risk factors for the presence of anxiety symptoms.

**Table 4:** Logistic regression results for sociodemographic characteristics associated with anxiety symptoms.

Variable	Overall		Urban		Rural	
	OR	95%CI	OR	95%CI	OR	95%CI
Gender						
Male	1		1		1	
Female	1.124	(0.966, 1.309)	1.052	(0.828, 1.337)	1.179	(0.968, 1.437)
Religious belief						
Yes	1		1		1	
No	1.027	(0.765, 1.380)	1.180	(0.667, 2.088)	0.967	(0.674, 1.366)
Class ranking						
<25.0%	1		1		1	
25.0%~<50.0%	0.871	(0.733, 1.036)	0.851	(0.642, 1.128)	0.884	(0.710, 1.100)
50.0%~<75.0%	0.801*	(0.664, 0.965)	0.808	(0.591, 1.104)	0.798	(0.631, 1.008)
≥75.0%	0.790	(0.693, 1.154)	0.759	(0.500, 1.152)	0.988	(0.716, 1.365)
Monthly per capita household income						
<2 500RMB	1		1		1	
2 500~5 000RMB	0.870	(0.722, 1.048)	0.800	(0.516, 1.239)	0.882	(0.716, 1.086)
5 000~10 000RMB	0.730**	(0.596, 0.893)	0.585*	(0.380, 0.901)	0.854	(0.664, 1.098)
≥10 000RMB	0.746*	(0.576, 0.964)	0.722	(0.456, 1.144)	0.658	(0.431, 1.005)
Exercise						
Yes	1		1		1	
No	1.018	(0.875, 1.184)	1.342*	(1.037, 1.738)	0.871	(0.721, 1.052)
Smoking						
Yes	1		1		1	
No	0.656	(0.429, 1.003)	0.770	(0.398, 1.489)	0.572*	(0.329, 0.993)
Drinking						
Yes	1		1		1	
No	0.905	(0.749, 1.093)	0.850	(0.642, 1.125)	0.944	(0.729, 1.223)
PHQ-9						
≥5	1		1		1	
<5	0.022***	(0.019, 0.026)	0.021***	(0.017, 0.028)	0.022***	(0.018, 0.027)
eHealth literacy						
≥32	1		1		1	
<32	1.159*	(1.001, 1.341)	1.097	(0.869, 1.386)	1.193	(0.986, 1.442)

*p<0.05, **p<0.01, ***p<0.001.

eHEAL, SeHealth Literacy Scale; GAD-7, 7-item Generalized Anxiety Disorder; PHQ-9, 9-item Patient Health Questionnaire.

In short, whether there is a difference in anxiety between urban and rural college students is mainly manifested in two key areas. First, per capita monthly household monthly income (5 000~10 000RMB, OR = 0.585) was identified as a risk factor only in the urban environment, while exercise only showed protective effects in the urban environment (NO, OR = 1.342). Second, smoking (NO, OR = 0.572) was a risk factor only in the rural setting.

### Decomposition analysis results

To ensure the stability of the results, the decomposition model was repeated 100 times using software. **Table 5** shows the results of the decomposition model of the differences between urban and rural college students in terms of the presence or absence of anxiety symptoms. The results showed that 88.42% of the observed factors contributed to the difference in the presence or absence of anxiety symptoms, while 11.58% were due to urban and rural factors and no observed factors. The results showed that the factors that contributed significantly to the difference in the presence or absence of anxiety symptoms (*P*<.05) included PHQ-9 scores (51.07%) and exercise (7.07%).

**Table 5:** The Fairlie decomposition model of anxiety symptom status in urban and rural university students.

Terms of decomposition			Anxiety symptom status	
Difference			-0.033	
Explained (%)			-0.030 (88.42)	
Non-explained (%)			-0.003 (11.58)	
Contribution to differences	B	P	Contribution (%)	[95%CI]
Explained				
Gender	-0.0005636	0.619	1.67	(-0.0027845,0.0016572)
Religious belief	-0.0004138	0.525	1.23	(-0.0001244,0.0010584)
Ranking of results	0.000467	0.122	-1.38	(-0.0016881,0.0008606)
Monthly per capita household income	-0.0094206	0.066	27.91	(-0.0194764,0.0006352)
Exercise	-0.0023856*	0.021	7.07	(-0.0044048,-0.0003664)
Smoking	0.0002467	0.299	-0.73	(-0.0002191,0.0007124)
Drinking	0.0013692	0.270	-4.06	(-0.0010648,0.0038032)
PHQ-9	-0.0172401	<0.001	51.07	(-0.0181476,-0.0163326)
E-health literacy	-0.0016535	0.436	4.90	(-0.0058102,0.0025033)

*p<0.05.

eHEAL, SeHealth Literacy Scale; GAD-7, 7-item Generalized Anxiety Disorder; PHQ-9, 9-item Patient Health Questionnaire.

## Discussion

The purpose of this study was to explore the differences in anxiety symptoms between urban and rural Chinese college students and to quantify the contribution of various types of factors to this difference. The results of the study indicate that there is a significant difference in the prevalence of anxiety symptoms between urban and rural college students, and that this difference is influenced by a variety of factors. By analysing these factors, we can not only reveal the distribution of anxiety symptoms among urban and rural college students, but also provide a theoretical basis for further interventions.

This study found that 38.91% of college students had anxiety symptoms, and there was a significant difference in the incidence of anxiety symptoms between urban and rural college students. Specifically, the prevalence of anxiety symptoms among rural college students was 40.2%, which was significantly higher than the 36.8% of urban college students. This result is consistent with other studies in China, indicating that there is a significant gap between urban and rural college students in terms of mental health (35). The higher prevalence of anxiety symptoms among rural college students compared with the national college student population highlights the prominent problems in mental health in rural areas (36). This unequal mental health situation is further associated with differences in resources, education, and social support networks between urban and rural areas, especially in the context of rapid changes in modern society (37). Additionally, disparities in mental health infrastructure between urban and rural regions have been shown to exacerbate these issues. Rural areas often face a shortage of mental health professionals, facilities, and specialized services, which significantly limits access to mental health care. Studies have highlighted that rural students are less likely to receive adequate mental health services, which can worsen psychological conditions such as anxiety and depression (38). The lack of adequate mental health services in rural areas is compounded by a shortage of qualified mental health professionals, such as psychologists and counselors, as well as insufficient community-based mental health programs. This gap in infrastructure leaves many rural students without access to timely psychological support, increasing the risk of untreated anxiety and depression (39). Research has shown that rural students often face difficulties in seeking mental health services, which are often located far from their homes and are difficult to access due to transportation and cost barriers (40).

Differences in anxiety symptoms among rural college students were largely associated with factors such as family income, exercise habits, and depressive symptoms (41, 42). Income showed different patterns across urban and rural settings. Among urban college students, those from upper-middle income households (5,000–10,000 RMB) were significantly less likely to report anxiety symptoms compared with the lowest income group (<2,500 RMB), suggesting a protective effect. In contrast, in rural areas, the association between income and anxiety was less linear and did not reach statistical significance. However, descriptive results suggested that students from middle-income households (2,500–5,000 RMB) may still face elevated psychological pressures compared to their peers from the lowest and highest income groups. Additionally, students who lacked exercise habits had significantly higher rates of anxiety symptoms. The study suggests that the impact of family income on mental health is not only material but also affects students’ social support networks and access to information. In contrast, in urban areas, although the effect of household income was smaller, other socio-economic and cultural factors, such as the quality of education, social support, and abundant access to information, clearly contribute to the alleviation of anxiety symptoms (43–45). While smoking did not significantly contribute to the observed differences in anxiety symptoms (P = 0.299, Contribution = −0.73%), it was retained in the model due to its theoretical relevance in influencing mental health as suggested by previous literature. Smoking has been shown to have an impact on mental health, with studies indicating its association with increased levels of anxiety and depression among various populations (46, 47). Although its effect was not significant in our study, future research could further investigate its role, particularly in specific subpopulations or under different conditions. Thus, the urban-rural difference is not only an economic issue, but it also involves a variety of factors such as social structure, educational resources, and cultural backgrounds (48–51).

This study found significant gender differences in anxiety symptoms between rural and urban college students. In rural Chinese universities, female students exhibited higher anxiety levels than their male counterparts. This disparity can be attributed to traditional gender expectations in rural settings (52, 53), where women face pressures related to academic success, family responsibilities, and social compliance. These gender roles often limit access to educational and mental health resources, increasing anxiety. Additionally, rural female students tend to experience more economic stress, as many come from lower-income families, and have lower participation in physical activities, which are known to alleviate anxiety (54). In contrast, urban female students also exhibit higher anxiety levels compared to their male peers. This may be due to societal pressures related to academic achievement (53, 55), career development, and societal expectations regarding marriage and family. Despite having better access to mental health resources, the compounded stress from these pressures may still exacerbate anxiety. Interestingly, rural male students exhibited lower anxiety levels than urban female students. This might be due to lower societal expectations on rural males in comparison to urban females, particularly in academic and professional domains. However, rural males still face significant stress from economic and family responsibilities. While these pressures may not manifest as overt anxiety, rural males are likely coping with these stresses in more subdued ways. Therefore, the lower anxiety levels observed in rural males may reflect different coping mechanisms and social expectations rather than a lack of stress.

Using logistic regression analyses, we found that academic performance (measured by class ranking in this study), exercise habits, and PHQ-9 scores all had a significant impact on anxiety symptoms among urban and rural college students. Better academic performance emerged as a protective factor for reducing anxiety symptoms, which is consistent with previous research (56). Higher academic achievement is typically associated with better information acquisition and coping skills, suggesting that academic achievement may reflect college students’ greater ability to cope with psychological stress. In addition, exercise habits, as a general protective factor, are effective in reducing the occurrence of anxiety symptoms. Exercise not only enhances an individual’s sense of health, but also promotes social interaction and information acquisition, which can help alleviate anxiety (57, 58). Exercise habits not only improve students’ physical fitness and health, but also help them maintain a positive mindset in the face of academic stress by promoting biological mechanisms such as endorphin secretion. The negative correlation between exercise and anxiety has been demonstrated in both urban and rural college students, especially in urban college students, where good exercise habits may play an important role in alleviating anxiety symptoms (59). However, the lack of infrastructure and public resources in rural areas may lead to lower participation in sport as students grow up and the protective effects of sport are not fully realised.

Compounding these structural gaps, our study also reveals a significant urban-rural divide in eHealth literacy—a critical competency in the digital age. Only 37.1% of rural students met the threshold for adequate eHealth literacy (≥32 on eHEALS), compared to 52.3% of urban peers (P<0.001). This 15.2% disparity reflects broader inequities in digital access, technology training, and health information-seeking behaviors in rural China (60). Although its contribution was not statistically significant in decomposition analysis (p=0.436), the real-world implications suggest that the urban-rural divide in eHealth literacy may exacerbate barriers to mental health help-seeking, particularly in rural areas where in-person services are scarce (61). This aligns with studies showing that low eHealth literacy correlates with poorer self-management of psychological symptoms and reduced engagement with teletherapy platforms (41).

This study also found that gender showed different effects on the occurrence of anxiety symptoms in urban and rural college students. In rural areas, the prevalence of anxiety symptoms was significantly higher among female college students than among males, which may be related to the persistent gender education gap and traditional social role concepts in rural areas (62). In rural China, females typically face more constraints in accessing education and social resources, resulting in greater stress on their mental health (63). These gender differences suggest that gender-specific needs should be taken into account when developing mental health interventions, and that intervention programmes should be tailored. In contrast, urban university students showed less gender differences, possibly due to a more egalitarian social environment and richer resource support in cities (64). Educational resources, information dissemination, and health management systems are more robust in cities, which helps urban college students mitigate the negative impact of gender factors on mental health as they grow up. The different manifestations of urban-rural differences in gender highlight the importance of gender equality in mental health interventions (65). In addition, gender role expectations and pressures in the cultural context have a profound impact on rural women’s mental health (66–68).

This study also found that family income played an important role in the differences in anxiety symptoms between urban and rural college students. In rural areas, students with lower monthly family incomes faced a higher risk of anxiety, reflecting the psychological burden of financial hardship and the lack of supportive resources (69). Interestingly, in urban areas, students from households with a per capita monthly income of 5,000–10,000 RMB showed a significantly lower risk of anxiety compared with those from the lowest income group (<2,500 RMB). This protective effect may be explained by the relative stability and adequate resource availability of upper-middle-income families, which can buffer against stress and provide access to supportive networks. By contrast, the lowest income group is more likely to experience financial insecurity and insufficient access to mental health resources, while students from the highest income families may also encounter unique pressures such as heightened academic or career expectations. These findings suggest that the relationship between income and mental health may not be linear but rather context-dependent, differing across socioeconomic settings and between urban and rural environments (70).

This study also found that PHQ-9 scores were highly correlated with anxiety symptoms. Depressive symptoms are strongly associated with anxiety symptoms, and chronic depression may affects an individual’s cognitive functioning and emotion regulation, and may reduce their ability to understand and process health information (71, 72). Among urban and rural college students, those with higher PHQ-9 scores are typically at higher risk for anxiety symptoms, a finding that emphasises the need to focus on the management of depressive symptoms alongside interventions for anxiety symptoms in college students. Co-morbidity of depression and anxiety may exacerbate an individual’s mental health problems; therefore, comprehensive intervention strategies should incorporate both to achieve the best treatment outcomes (73).

This study further analysed the sources of anxiety symptom differences between urban and rural college students using the Fairlie decomposition model. The results showed that PHQ-9 scores and exercise habits contributed the most to the difference in anxiety symptoms, with 51.07% and 7.07%, respectively. The finding that depressive symptoms were associated with more than half of the anxiety disparity highlights the importance of addressing depression-anxiety comorbidity in interventions targeting anxiety among college students. Given the significant explanatory power of depressive symptoms, integrating routine depression screening and targeted mental health support for depressive symptoms into existing anxiety intervention programs could substantially enhance their effectiveness. This result not only helps us to better understand the mechanism of anxiety symptom generation, but also provides empirical evidence for designing targeted intervention strategies.

In order to effectively narrow the gap between urban and rural university students in terms of mental health, this study makes a number of policy recommendations. First, mental health education should be strengthened, especially mental health support for students from rural areas. Although many rural-origin college students go to the city to study, they are still affected by factors such as the environment of their place of origin, their family’s economic situation, and their society and culture. Therefore, colleges and universities should provide customised mental health education courses for these students to help them better adapt to university life and cope with academic pressure and emotional distress (74). Course content should include psychological adjustment skills, emotion management and stress coping strategies, especially how to maintain psychological balance when facing changes in urban and rural cultures and environments. Second, increase the construction of online mental health support platforms. Many rural-born college students come from relatively economically underdeveloped areas and may lack timely professional help during their growth (75). To make up for this shortcoming, it is recommended that the state or universities ensure that both urban and rural students have equal access to professional psychological support by strengthening the construction of online mental health platforms that provide services such as online psychological counselling, webinars and popular science articles on mental health. These platforms can take the form of video calls, text counselling and psychological assessment tools to help students access psychological help even in non-campus environments, especially during holidays such as winter and summer breaks. However, recognizing infrastructure limitations in rural areas, hybrid delivery models combining both online and offline resources could be adopted, particular attention should be paid to the gap in e-health literacy among rural students. For instance, considering unstable internet access in rural regions, universities could develop mental health support materials accessible via mobile networks, such as short messaging services (SMS) or offline-compatible mobile applications. Additionally, printed mental health educational materials could be regularly distributed through partnerships with local community centers or rural health clinics. Universities might also cooperate with local governments or community health institutions to establish periodic mobile mental health clinics, offering face-to-face psychological counseling for rural-origin students during academic breaks. Promoting social support networks is also part of effective intervention. Compared with urban college students, rural-born college students are usually weaker in social support networks. For this reason, schools should establish a multi-level support system and encourage students to participate in various social activities and groups, such as psychological clubs and interest groups. Providing rich group activities on campus, such as regular social gatherings, sports events, and volunteer activities, helps rural students establish a sense of belonging, promotes their interactions with classmates and teachers, and enhances their emotional support network and perceptions of social support, thereby alleviating anxiety symptoms. In addition, exercise intervention should be used as one of the core strategies to alleviate college students’ psychological stress and anxiety symptoms (76). For urban and rural college students, exercise has been an effective way to relieve psychological stress and anxiety symptoms. Schools can motivate students to engage in more physical activity through the improvement of school sports facilities and the diversification of sports programmes. To address the lack of sports facilities in rural areas, universities could collaborate closely with local community organizations to identify and utilize existing spaces, such as local schools, community centers, and parks, to hold regular physical activities. Temporary or mobile sports facilities and equipment could also be provided through university-community partnerships, ensuring rural students have equitable access to physical exercise opportunities. Such collaboration could effectively mitigate infrastructure constraints and facilitate implementation of exercise-based mental health interventions. To this end, schools can cooperate with local governments and social organisations to promote the integration of community sports activities and college student fitness programmes, encouraging students to maintain a healthy body and a positive mindset outside of academia. Finally, the precise intervention of gender differences should not be ignored. Due to the socialisation of gender roles, female students face more pressure on their mental health, especially in rural areas where traditional gender role expectations may exacerbate women’s psychological distress (77, 78). Therefore, schools should implement special mental health interventions for female students. For example, specialised female mental health lectures and workshops should be offered to help female students cope with academic challenges, social pressures and gender role conflicts, and to improve their mental resilience and coping skills. In addition, in the process of psychological counselling, counsellors and psychologists should pay special attention to the emotional needs and psychological stress of female students and provide personalised support.

## Limitations

This study has several limitations that warrant consideration. First, due to the cross-sectional design, we are unable to establish temporal or causal relationships between anxiety symptoms and the explanatory factors, including depressive symptoms (PHQ-9), exercise habits, and eHealth literacy. The Fairlie decomposition results represent statistical contributions to group differences rather than directional causality. Second, all variables were measured using self-reported questionnaires, including GAD-7 and PHQ-9, which may introduce recall bias or social desirability effects. Third, due to the limitations of the original survey design, several theoretically relevant variables—such as academic stress, perceived social support, and peer relationships—were not included. These unmeasured factors may also influence the observed differences in anxiety symptoms and should be examined in future research. Lastly, although our sample was drawn from ten provinces with diverse geographic coverage, it may not fully represent all university students in China, especially those from ethnic minority regions or remote rural areas. In addition, regional variability between different geographic areas of China (e.g., coastal vs. inland regions) was not explicitly addressed in our analysis. Regional differences in socioeconomic development, educational resources, and cultural contexts may influence anxiety disparities between urban and rural students. Therefore, caution should be exercised when generalizing the findings nationwide. Future research should explicitly incorporate regional variables into the analysis to better understand how geographic contexts impact mental health disparities. Longitudinal studies with broader variable coverage and representative sampling are needed to strengthen the generalizability and causal interpretation of these findings.

## Conclusion

This study reveals significant differences in anxiety symptoms between urban and rural college students and quantifies the contribution of various factors to such differences through deconstructive analyses. The findings provide valuable insights for policy makers and educators, suggesting that bridging the urban-rural mental health gap requires not only a focus on traditional mental health interventions, but also a combination of socio-economic and cultural factors and modern technological tools to provide comprehensive and personalised support for university students. By systematically analysing these influencing factors, we will not only be able to better understand the root causes of the differences in anxiety symptoms between urban and rural areas, but will also be able to provide a solid empirical basis for the development of effective interventions.

## Data Availability

The raw data supporting the conclusions of this article will be made available by the authors, without undue reservation.
